# Colica Pictonum

**Published:** 1848-09

**Authors:** 


*Colica Pictonum.* By JAMES H. JOHNSON, M.D., one of the Physicians and Surgeons of the "St. Louis Hotel for Invalids."

The pathology and treatment of Colica Pictonum, has for many years invited the attention and excited the minds of medical men. Its diagnostic similarity to other varieties of enteritic inflammation, and spasmodic intestinal irritation, have served to mislead the profession, from what I conceive to be the true cause of the disease, and consequently has indirectly produced many errors in practice.

This article is not written with a view to explain all the physiological derangements, or abnormal appearances of the intestinal tube, but with a design to recommend a system of practice, which has proved generally successful in my hands, for a number of years, in the mining regions of northern Illinois, Wisconsin, and Iowa.

The following remarks are presented to the profession with some confidence, as the theory is based upon clinical observations, and post mortem examinations. I do not wish to assume, or claim any principle as new in Pathology, but to only insist upon the active indications of cure.

It is useless for me to enter into the general symtomato!ogy of the disease, as every medical man is presumed to understand its peculiar and various phases.

The disease depends upon the spasmodic action of the smaller intestines; the jejunum and ilium, and frequently extends to the larger portion of the canal as far as the sigmoid flexure of the colon. I have seen gangrenous erosions, which had been treated by clever men in the profession, as cases of intussusceptio, which erosions can only be accounted for upon the principle that a compression upon the spinal marrow produces a paralysis of the peristaltic action of the intestinal canal in consequence of the sensorial fluid from some of the vertebral nerves being obstructed in its course. Hence, a change, of circulation followed by gangrene. I would invite the attention of the practitioner to the peculiar expression, and langour of the countenance of the patient. The warm chest, yet, cold extremities, the glassy eye, milk furred, yet dry-pointed tongue, which, in connection with the contraction of the abdominal muscles, the palsied and drooping hand, and contracted fingers, and faint efforts at vomiting, are the best pathognomonic symptoms of the disease.

In reference to the exciting cause, I will speak from observation, discarding many of the opinions entertained by distinguished gentlemen of the profession. Among the smelters in the mining regions mentioned, the disease is not from an Arcinite (as has been supposed,) which is so minutely divided as to impregnate the atmosphere in the neighborhood of the furnaces. The effluvium, always laying the foundation for the disease. I will also affirm that lead, or preparations of lead, in the form of carbonate, frequently produces paralysis, general neuralgic symptoms, and in one case to knowledge, amaurosis, with all the concomitant symptoms, depending upon derangement of the cerebro-spinal axis, and that particular portion of the brain, the thalami nervorum opticorum, from which the optic nerve originates. This case was owing to the use of the "cosmetics of the shops," which is a fine preparation of the carbonate of lead.

I will here assert, that the remote cause of Colica Pictonum, and Rhachialgia, in a majority of cases, appears to be lead introduced into the system, through the stomach, the lungs, or the skin. Hence, the disease is most frequently found in the neighborhood of the smelter's furnace, and the probability is, that the disease is more apt to occur from the fumes of the furnace, taken into the lungs, than from endermic or stomachic absorption. In the vicinity of the furnaces at Galena, animals, such as horses, oxen, pigs, and even poultry, are subject to the disease. The operative miners and citizens, generally, are under the belief that the exciting cause depends upon the water drank from the streams running below the wash dams, &c. This is a popular error—pure water does not act upon the sulphuret of lead, consequently it is harmless. But lead is easily disintegrated and reduced to an oxyde, in the process of smelting, and exists in the atmosphere; thus, we may readily conceive why man and beast are alike subject to the same disorder. Spring or river water is incapable of decomposing the sulphuret of lead in sufficient abundance to form either an oxyde or any of the salts that would prove injurious to health.

Another error exists, not only in the minds of the people, but frequently with the practitioner, "that fatty, or oily food is a preventive and cure of the disease,"—as a prophylactic, it may be of service, but as remedial agents, none of the oleaginous preparations can be depended upon.

It is true, these agents may be indirectly serviceable in ordinary cases, but the direct indications of cure are more active. My practice is laid on the firm and solid foundation of experience and observation—an experience of several years—with every opportunity of close investigation, so far as regards the pathology of the disease, and the beneficial results derived from the following treatment:

In the first place, counteract by all possible means the intestinal spasm; this is best done by large doses of proto. chl. hyd. and opium, say from 30 to 50 grs. of the former combined with from 2 to 5 grs. of the latter, to be repeated at least once in three hours. In some cases opium may be given much oftener, as no idiosyncrasy ever occurs in cases of colica pictonum. In two hours after the last dose of calomel and opium, give from 2 to 4oz. of ol. ricini combined with from 4 to 5 gtt. of ol. tiglii. Generally, in a few minutes, a copious and free evacuation will take place; if not, then commence injections, not a pint, or a quart, nor a gallon, but enough to distend the bowels. It sometimes may require a large quantity of fluid—but continue; the object is, or should be distension—nature then will perform her function. Many eminent practitioners prescribe injections for the purpose of softening the intestinal fœcal matter, and for increasing the peristaltic motion of the bowels. The true principle in colica pictonum is to *distend*, and consequently to remove the intestinal spasm.

In a mild degree, and under the best therapeutic plan, the disease can seldom be removed under four or five days, but if it be neglected or maltreated, it may continue for weeks, months, or even years, and then will terminate either in palsy of the upper extremities, depending, as before remarked, upon derangement of the cerebro spinal axis, partial, or complete amaurosis, deafness, delirium, or epileptic fits, and thus continue until death closes the eyes of the sufferer.

After the primary practice recommended, as a neutralizing agent, I would mention the use of the sulphureted hydrogen gas. I have used it in many cases with decided advantage, and in fact, believe it to be the only remedy worthy the attention of the practitioner, in such forms of diseased action, where the patient is not at once restored to comparative health, subsequent to the acute attack passing off.

It should be used in the form of a lemonade, the patient drinking from three to five common sized tumblers full every day. I am indebted to Dr. Henry King, a practical and scientific chemist of this city, for the following formula, which he considers the most easy and convenient method of preparing, as all the materials are within the reach of every physician, either in town or country.

Prepare *sulphuret of iron* by rubbing a stick of sulphur on a hot bar of iron, and collect the metal materials, or by mixing flour of sulphur and iron filings, in the proportions of sixteen ounces of the latter with ten and a half of the former, and fusing them together in an earthen crucible. This should be done by putting the mixed materials into the crucible in small portions at a time, and allowing that which has been thrown in to become red hot before another portion is added.

Add diluted sulphuric acid with about four times its volume of water, and allow it to cool. Place a small quantity of the sulphuret in a vial or bottle with a cork perforated with a small glass tube bent into the form of a syphon. When the gas begins to be disengaged, insert the free end of the glass tube into a vessel of water, which will soon become saturated with the gas. If it be desirable to keep the water for some time, this operation should be performed in four or eight ounce vials, which should be subsequently carefully corked and inverted.

Sulphureted hydrogen water may also be made by placing the sulphuret of iron and diluted acid in a large bottle, and adding water until the whole is largely diluted. But in this case a portion of the salt formed (sulphate of the prot-oxide of iron,) will be dissolved in the fluid.

The diluted sulphuric acid is a valuable remedy in cases where the sulphureted hydrogen cannot be made or procured. Give it in large doses, repeated several times a day.—*St. Louis Medical and Surgical Journal.*

